# Bullöses Pemphigoid bei „skin of color“ – klinisches Bild der perifollikulären Melanozytenregeneration im Rahmen der Reepithelisierung

**DOI:** 10.1007/s00105-025-05518-9

**Published:** 2025-06-06

**Authors:** Can Raul Alpagut, António Manuel Sequeira Santos, Michael Hertl, Martin Gschnell

**Affiliations:** Universitätsklinikum Marburg, Klinik für Dermatologie und Allergologie, Philipps-Universität Marburg, Baldingerstr., 35043 Marburg, Deutschland

## Anamnese

Wir berichten über einen 84-jährigen Patienten mit Hauttyp VI nach Fitzpatrick und eritreischer Herkunft ohne Nebendiagnosen oder dermatologische Vorerkrankungen, der sich mit Hämatemesis sowie einem erosiv-bullösen Hautbild vorstellte. Die Symptome bestünden bereits seit 6 Monaten. Der Patient klagte über starken Juckreiz an der Haut sowie über regelmäßigen blutig tangierten Sputumauswurf einhergehend mit starken oralen Schmerzen insbesondere bei der Nahrungsaufnahme.

## Untersuchung

**Abb. 1:**
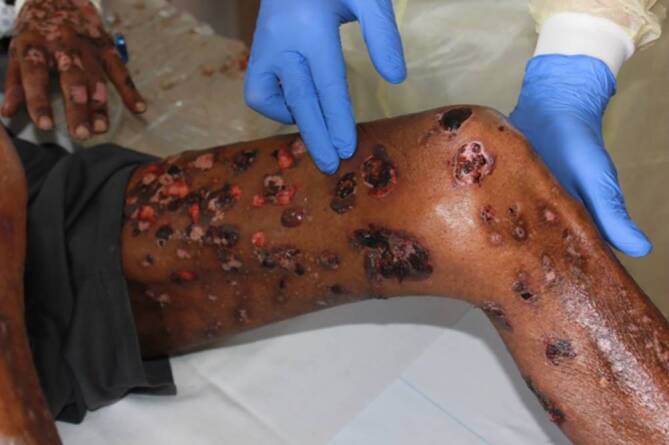
Klinischer Befund bei Erstvorstellung: Es zeigen sich exemplarisch am linken Handrücken sowie am linken Ober- und Unterschenkel ein Mischbild aus Erosionen, prallen dunklen Blasen sowie Hypo- und Hyperpigmentierungen

Die Haut des Patienten wies am gesamten Integument einschließlich des Rumpfes Erosionen, pralle Blasen, hämorrhagische Krusten sowie Hypo- und Hyperpigmentierungen auf (Abb. 1). Die Mundschleimhaut war im Bereich des harten Gaumens erodiert. Ober- und Unterlippe wiesen ebenfalls Erosionen auf, während die Genitalschleimhaut nicht betroffen war.

## Diagnostik

Eine Gastroskopie ergab eine schwere erosive Ösophagitis ohne Zeichen einer aktiven Blutung. Die entnommene Histologie sowohl an der Haut am Abdomen sowie aus dem Ösophagus zeigte eine fokale Spaltbildung supepidermal mit überwiegend lymphozytärem Infiltrat mit perivaskulärer Akzentuierung sowie zahlreichen eosinophilen Granulozyten im Interstitium. Mit der direkten Immunfluoreszenz aus der Haut konnten IgG-Ablagerungen am Blasendach und C3-Ablagerungen am Blasendach und Blasenboden nachgewiesen werden. In der indirekten Immunfluoreszenz waren lineare Ablagerungen entlang der Basalmembran (Affenösophagus) und IgG-Ablagerungen am Blasendach (Kochsalzspalthaut) positiv. Außerdem waren die Antikörper im Serum mit BP-180 (2133 RE/ml) positiv. BP-230- sowie Laminin-332-Antikörper waren negativ. In der Zusammenschau der Befunde konnte die Diagnose eines bullösen Pemphigoids mit Schleimhautbeteiligung gestellt werden.

## Therapie und Verlauf

**Abb. 2:**
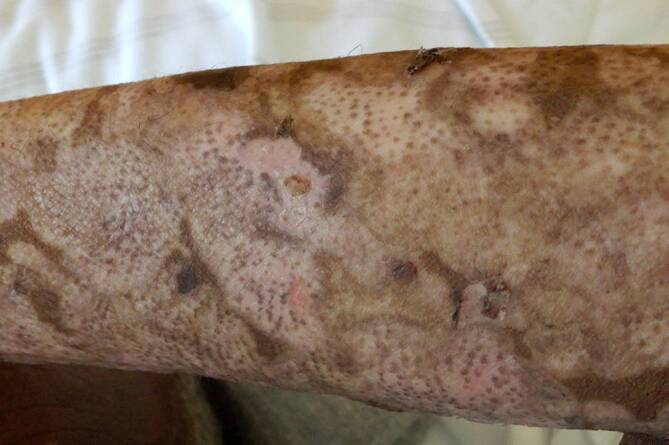
Klinischer Befund 1 Woche nach Therapiebeginn: Es zeigt sich die follikulär betonte Repigmentierung innerhalb der ehemals erodierten Blasen am Unterarm

Es erfolgte eine lokale Therapie mit einer Clobetasol 17-propionat-haltigen Creme 2‑mal täglich. Systemisch wurde eine Stoßtherapie mit 100 mg Dexamethason intravenös für 3 Tage eingeleitet und anschließend auf Prednisolon 0,5 mg/kg Körpergewicht oralisiert. Bei laborchemisch erhöhten Infektparametern erfolgte neben der i‑v.-Antibiose mit Ceftriaxon zudem die Einleitung einer Therapie mit Doxycyclin 100 mg per os 2‑mal täglich. Supportiv erhielt der Patient im Bereich der Mundschleimhaut eine Therapie mit Ampho-Moronal-Suspension und einer Lidocain-haltigen Lösung sowie eine moderne Wundtherapie für das betroffene Integument. Unter der eingeleiteten Therapie zeigte sich eine Besserung der Hautbefundes mit Tendenz zur Reepithelisierung. Das Hautbild zeichnete sich durch ein besonders eindrucksvolles Repigmentierungsmuster aus. In den hypopigmentierten Arealen im Bereich der zuvor stattgefundenen epidermalen Ablösung konnten follikulär betonte dunkle Makulae beobachtet werden (Abb. 2). Der Patient war während des gesamten Aufenthaltes nicht der UV-Strahlung exponiert.

## Diskussion

Wie im vorliegenden Fallbericht beim Hauttyp VI nach Fitzpatrick sind die dunklen Makulae durch Repigmentierungen der Haut gekennzeichnet, die sich zunächst punktförmig aus den Haarfollikeln gebildet und im Verlauf auf das gesamte Hautareal ausgebreitet haben. Dies korreliert mit der Defekttiefe der Haut. Beim bullösen Pemphigoid gehen mit der Ablösung der Epidermis auch die Melanozyten verloren, da diese im Stratum basale der Epidermis direkt an der Basalmembran liegen [2]. Die Stammzellen der Melanozyten sind unter anderem in den Haarfollikeln lokalisiert [7]. Follikuläre Stammzellen der Melanozyten wandern nach Verletzungen in einem Melanocortin-1-Rezeptor-abhängigen Prozess in die Epidermis ein, wo sie sich zu funktionellen Melanozyten differenzieren und die epidermale Melaninproduktion übernehmen [4]. Das Repigmentierungsmuster lässt sich durch die perifollikuläre Regeneration von Melanozyten oder durch die Migration von Melanozyten aus den Haarfollikeln erklären. Bei einer intraepidermalen Spaltbildung hingegen, wie sie z. B. beim Pemphigus vulgaris auftritt, ist eine kontinuierliche, flächigere Repigmentierung zu erwarten. Dabei ist jedoch klar zu betonen, dass sich dieses Repigmentierungsmuster nicht nur bei dunklen Hauttypen finden lässt, sondern hier aufgrund der dunklen Pigmente besser erkennbar ist. Solche klinischen Unterschiede bei Patienten und Patientinnen mit „skin of color“ können dem Untersucher Hinweise auf die Defekttiefe geben. Dieser Informationsgewinn ist nicht nur auf bullöse Autoimmundermatosen beschränkt. Die aktuelle Literatur bietet im deutschsprachigen Raum nicht ausreichend Bildmaterial zur Diagnostik und Therapie von Dermatosen bei „skin of color“ [5]. Eine Erweiterung der Literatur mit Bildmaterial sowie Fallberichten bei „skin of color“ ist unerlässlich. Ein verstärktes Fortbildungs- und Literaturangebot könnte Dermatologinnen und Dermatologen in Zukunft helfen, solche Hautpräsentationen, wie oben beschrieben, gezielt wahrzunehmen und somit sicherere und frühere Diagnosen zu stellen. Unser Fall unterstreicht das klinische Bild der perifollikulären Melanozytenregeneration im Sinne einer Repigmentierung, insbesondere bei Patient*innen mit dunklerem Hauttyp. Weitere Studien zur Erforschung der klinischen Manifestation von Hautkrankheiten bei „skin of color“ sowie deren Darstellung in der Aus- und Weiterbildung werden zunehmend notwendig [1, 6, 8]. Zusätzlich ist bei unserem Fall hervorzuheben, dass die ösophageale Beteiligung beim bullösen Pemphigoid selten ist und in etwa 4 % der Fälle auftritt. Die Beteiligung der Speiseröhre kann asymptomatisch sein oder sich mit Symptomen wie Dysphagie, Odynophagie, Brustschmerzen oder gastrointestinalen Blutungen manifestieren [3].

## Fazit für die Praxis

Das klinische Erscheinungsbild des Verteilungsmusters der Repigmentierung bei Patienten mit „skin of color“ lässt Rückschlüsse auf die Defekttiefe und damit auf die verschiedenen bullösen Autoimmundermatosen zu.
